# Multifocal Desmoplastic Infantile Ganglioglioma/Astrocytoma (DIA/DIG): An Institutional Series Report and a Clinical Summary of This Rare Tumor

**DOI:** 10.3389/fonc.2021.608129

**Published:** 2022-05-09

**Authors:** Qiguang Wang, Jinli Meng, Jian Cheng, Si Zhang, Xuhui Hui, Qiang Li, Wenke Liu, Yan Ju, Lin Sun

**Affiliations:** ^1^ Department of Neurosurgery, West China Hospital, Sichuan University, Chengdu, China; ^2^ Department of Radiology, Hospital of Chengdu Office of People’s Government of Tibetan Autonomous Region (Hospital C.T), Chengdu, China; ^3^ Huaxi MR Research Center (HMRRC), Functional and Molecular Imaging Key Laboratory of Sichuan Province, Department of Radiology, West China Hospital, Sichuan University, Chengdu, China; ^4^ School of Computer Science and Engineering, University of Electronic Science and Technology of China, Chengdu, China; ^5^ Medical Insurance Office, West China Hospital, Sichuan University, Chengdu, China

**Keywords:** Multiple DIA/DIGs, treatment, outcome, infant, clinical feature

## Abstract

**Aim:**

Multifocal desmoplastic infantile ganglioglioma/astrocytoma (DIA/DIG) has rarely been reported. Here, two cases have been presented, reviewing the literature and proposed treatment algorithms for this rare tumor.

**Patients and Methods:**

We report two patients diagnosed with multifocal DIA/DIGs in West China Hospital. In addition, a literature review was performed, in October 2019, on case reports of DIA/DIGs with multifocal lesions. The clinical and radiological features, treatment, and outcome of this rare disease were discussed.

**Results:**

DIA/DIGs with multifocal locations were rare, and only thirteen cases (including ours) had been reported. This series included 8 males and 5 females with a mean age of 31.4 ± 45.7 months (range, 3-144 months). The supratentorial hemisphere, suprasellar region, posterior cranial fossa, and spinal cord were frequently involved. Ten patients (76.9%) received surgical resection for the symptomatic lesions and three patients (23.1%) underwent biopsy. Seven patients received chemotherapy postoperatively. Six individuals had tumor recurrences during the follow-up period, while three patients had tumors that spontaneously regressed. Finally, two patients died of tumor progression and one patient died of respiratory insufficiency and hypothalamic dysfunction.

**Conclusions:**

Multifocal DIA/DIGs have more aggressive clinical behavior and poor outcome despite benign histology. DIA/DIGs should be included in the differential diagnosis of multifocal brain tumors in children. The mainstay of treatment is surgical resection; adjuvant treatment with chemotherapeutic drugs is unknown and requires additional research.

## Introduction

Desmoplastic infantile astrocytoma (DIA) and desmoplastic infantile ganglioglioma (DIG) are rare intracranial tumors in infants, and account for only 1.25% of all childhood brain tumors (1). They most commonly manifest as large cystic or solid well-defined supratentorial lesions in infants aged 1 to 24 months (2). The WHO classification (2016) of nervous system tumors categorized DIA and DIG together as one diagnosis, among grade 1 “neuronal and mixed neuronal-glial” entities (3). They frequently present as solitary cortical-surfacing neoplasms, gross total resection can always achieve a favorable outcome (4).

Only 11 cases of DIA/DIGs with multifocal intracranial or intraspinal tumors have been recorded in the English literature (5–11). Its natural history, diagnosis and management remain a dilemma because of the rarity. Here, based on the institutional and literature cases, the largest case series of multifocal DIA/DIGs have been presented, to summarize the clinical features, optimize the treatment and elaborate the nature of this rare disease.

## Patients and Methods

### Case Presentation

#### Patient 1

**Figure 1:**
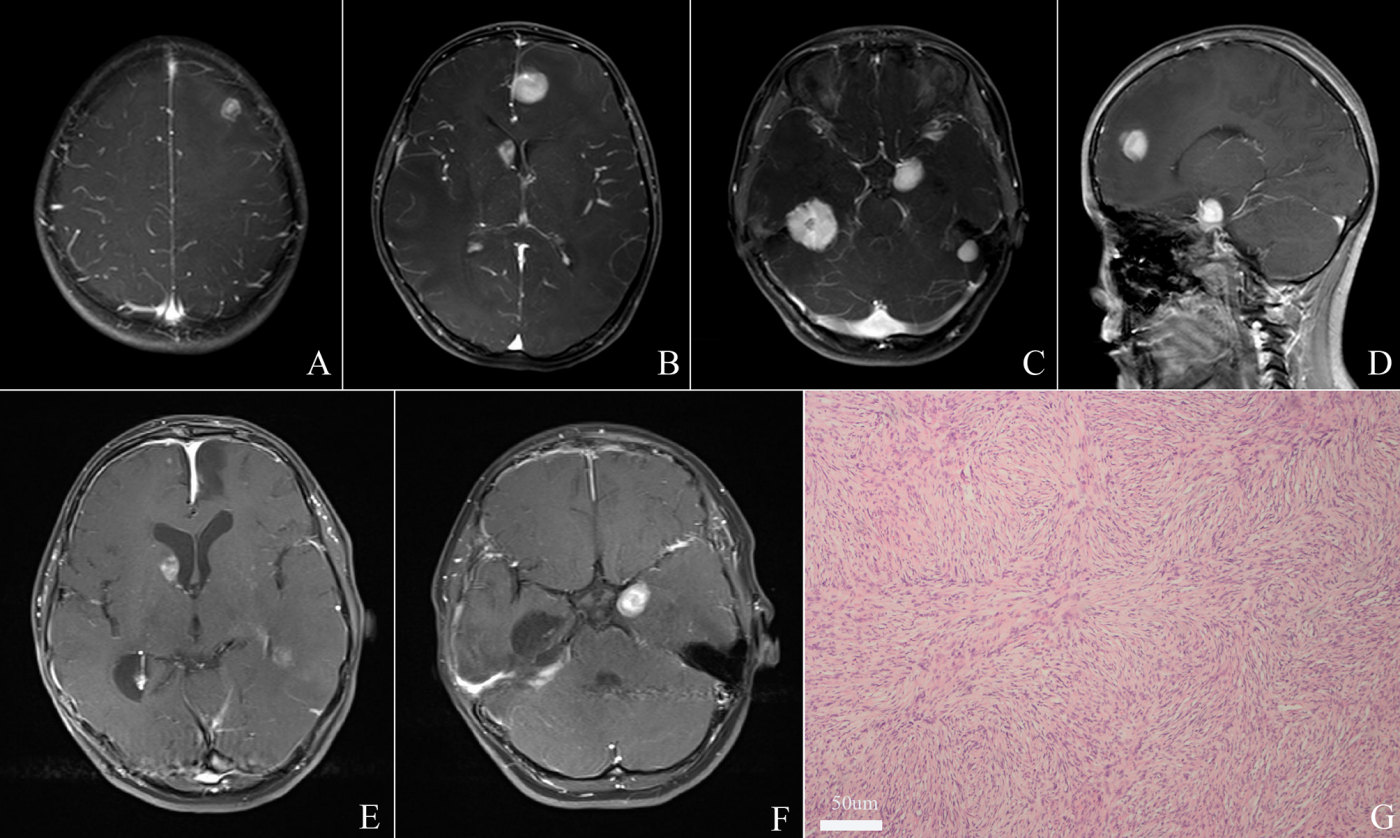
Cranial magnetic resonance imaging (MRI) depicted five lesions located in the right and left tentorium, left para sellar, right lateral ventricle, and left frontal lobe respectively **(A–D)**. Total resection of tumors in the left frontal lobe and right tentorium was achieved **(E, F)**. Histopathological examination revealed a desmoplastic spindle cell tumor with highly eosinophilic collagenous fiber, confirming the diagnosis of DIA/DIGs. (x 200) **(G)**.

A 12-year-old female was admitted to our hospital with seizures over two months. Cranial magnetic resonance imaging (MRI) depicted five solid tumors in the right and left tentorium, left para sellar, right lateral ventricle, and left frontal lobe respectively, the solid tumors are hypointense in T1 sequences and isodense or hypointense with obvious peritumoral edema, after administration of gadolinium, the tumors enhanced strongly (**Figures 1A–D**). She didn’t have a family history of genetic disease, but she had a previous surgery history of lung inflammatory myofibroblastic tumor. The tumors in the left frontal lobe and right tentorium were completely resected after admission to obtain a histopathological diagnosis and relieve symptoms **(** **Figures 1E, F** **)**. Postoperative histopathological examination supported the diagnosis of DIA/DIG **(** **Figure 1G** **)**. The patient was discharged from the hospital with a favorable outcome two weeks later and a close follow-up was achieved.

#### Patient 2

**Figure 2:**
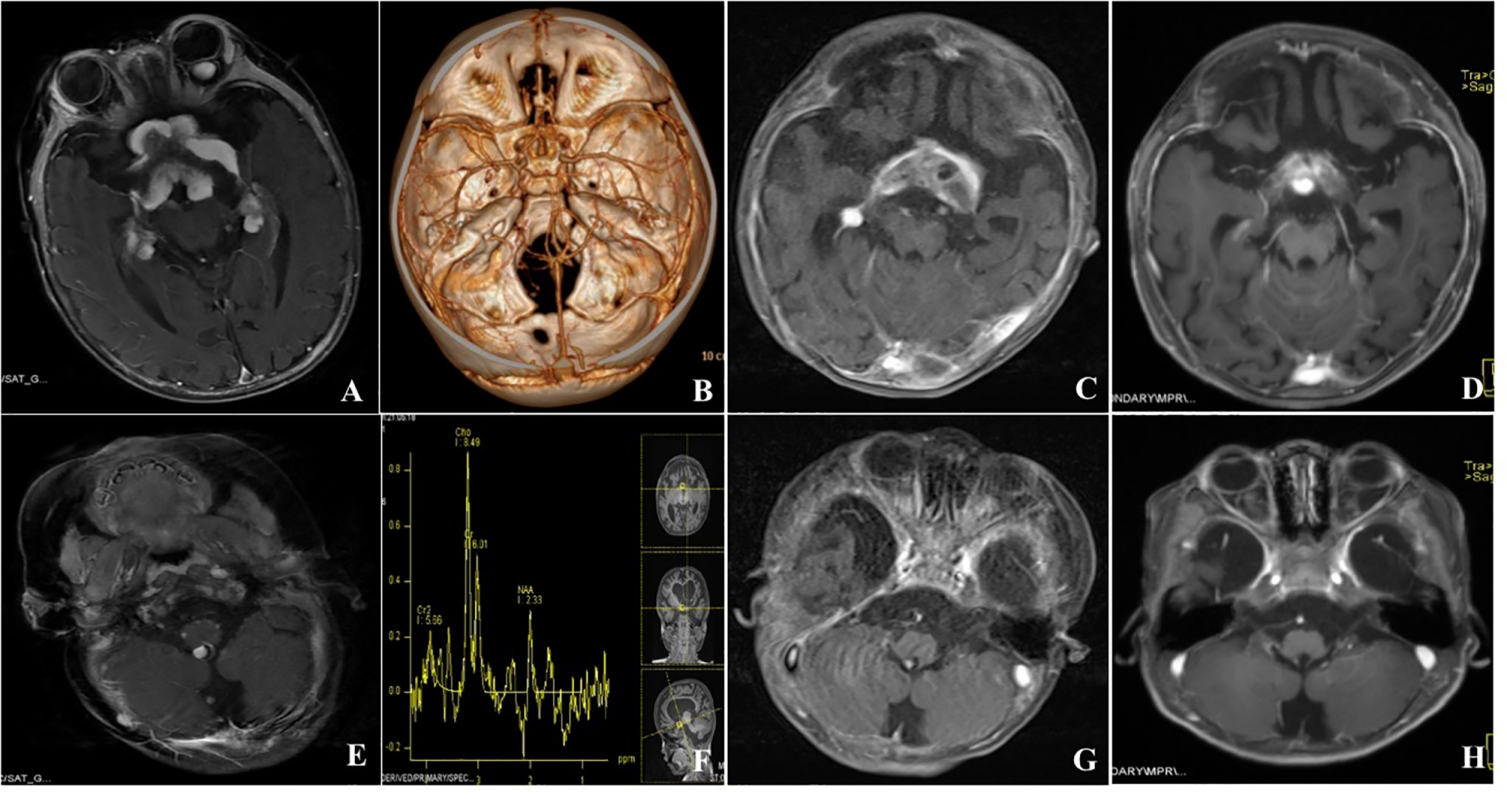
Gd-enhanced cranial MRI depicted multiple enhanced lesions in the suprasellar cistern, bilateral cavernous sinus and hippocampus, intraorbital and cisterna magna **(A, E)**. CT angiography showed that the lesions were not vascular tumors and no obvious vessels were found inside the tumor **(B)**, MR Spectroscopy showed that the lesion in the saddle area was featured by an elevated choline peak, a reduced NAA peak and a lactate peak **(F)**. Postoperative MRI depicted partial resection of suprasellar cistern tumors **(C, G)**. Further MRI was done at 22 months follow-up depicted spontaneous regression of the lesions in the medial hippocampus, suprasellar cistern, and cisterna magna **(D, H)**.

A 6-month-year old boy presented to our hospital with a history of bilateral nystagmus, exophthalmos for two months. Multiple lesions were seen on Gd-enhanced cranial MRI in the suprasellar cistern, bilateral cavernous sinus, and hippocampus, intraorbital, and cisterna magna (**Figures 2A, E** **)**. The suprasellar region tumors contained both solid and cystic components, the solid part showed hypointense in both T1 and T2 sequences, the cystic part showed hypointense on T1 sequences and hyperintense in T2. CT angiography depicted no obvious vessels inside the tumors (**Figure 2B**), MR Spectroscopy showed the tumor in the suprasellar was featured by an elevated choline peak, a reduced NAA peak, and a lactate peak (**Figure 2F**). He didn’t get the spinal MRI scan because he didn’t have any related symptoms. He didn’t have a family history of genetic disease. Due to the abundant blood supply and hard tumor texture intraoperatively, a subfrontal craniotomy was performed, and partial resection was obtained **(** **Figures 2C, G** **)**. Histologically, the tumor showed abundant collagenous fiber with fibroblastic and astrocytic elements. His parents chose regular follow-up without further adjuvant interventions. The follow-up MRI at 22 months depicted spontaneous regression of the lesions in the medial hippocampus, suprasellar cistern and cisterna magna (**Figures 2D, H** **)**.

### Literature Review

The data of patients with multifocal DIA/DIGs was collected according to the PRISMA (preferred reporting items for systematic reviews and meta-analyses) guidelines (12). The literature review was conducted on PUBMED/MEDLINE database with the following keywords: multiple, multifocal, metastases, desmoplastic infantile astrocytomas, desmoplastic infantile ganglioglioma. The search included only articles or case reports in English until October 2019 without an early date limit.

Case report or article titles and abstracts were evaluated. Only articles are written in English that described cases of multifocal desmoplastic infantile ganglioglioma/astrocytoma were included. The literature review was conducted independently by two authors (QG, W and J, C) using EndNote X7 software (Thomson Reuters, Carlsbad, California, USA). For controversial studies regarding the inclusion, a discussion would be performed until a consensus agreement was reached. The patient characteristics such as demographic data, clinical and radiological features, pathologic diagnosis, treatment and prognosis were extracted from the literature by two authors (QG, W and J, C) independently.

## Results

### Patient Sample, Clinical and Radiological Features

A total of 13 cases (including ours) **Table 1**) were included in the present study. The baseline data were summarized in **Table 2**. This series included eight infants with mean age of 5.7 ± 2.5 months (range, 3-11 months) and five children with average age of 6.2 ± 4.5 years (range, 1.2-12 years). Among them, 8 patients were male and 5 patients were female. The most common symptoms were those related to increased intracranial ICP or macrocephaly (n=5, 38.5%), nystagmus (n=3, 23.1%), seizures (n=4, 30.8%) and sensorimotor deficits (n=2, 15.4%). In this series, each patient had multiple tumors, and the multifocal lesions were found in the supratentorial hemisphere (n=8), suprasellar region (n=8), infratentorial location (n=8) and spinal cord(n=8), other locations include pial and ependymal, periventricular and tentorium. The lesions in first case from our institution were noted in tentorium, left para sellar, right lateral ventricle and left frontal lobe (**Figure 1**); the second case included tumors in the suprasellar cistern, bilateral cavernous sinus and hippocampus, intraorbital and cisterna magna (**Figure 2**). The solid lesions frequently showed iso- to hypointense on T1W sequences, predominantly hypointense on T2W sequences, and homogenous enhancement in Gd-enhanced MRI. Sometimes multifocal DIA/DIGs include both cystic and solid lesions, with the cystic component frequently being deep and the solid component being more peripheral; the cyst wall can be enhanced or not (8). In this series, the multifocal tumors were present at diagnosis in 12 cases, in the case presented by Darish et al (9), the multifocal leptomeningeal lesions were found six weeks after the initial diagnosis of DIA/DIGs.

**Table 1:** Patients' characteristics.

Characteristics	Number (%)
Cases	13
Gender	
Female	5 (38.5%)
Male	8 (61.5%)
Age, months (mean± SD)	31.4±45.7
Range	3-144
Number of tumors (mean± SD)	4
Range	3-7
Tumor Location	
Supratentorial hemisphere	8 (61.5%)
Suprasellar or parasellar region	8 (61.5%)
Infratentorial location	8 (61.5%)
Spinal cord	8 (61.5%)
Surgical resection (Surgical site)	
Gross total resection	5 (38.5%)
Subtotal resection	3 (23.1%)
Partial &Biopsy	5 (38.5%)
Follow-up period	
Months (mean ±SD)	16.9±16.5
Range	1-38
Outcome	
Dead	3 (23.1%)
Alive	10 (76.9%)
Chemotherapy	7 (53.8%)
Tumors progression	6 (46.2%)
Symptoms	
Macrocephaly	5 (38.5%)
Headache, vomiting, increased intracranial ICP	4 (30.8%)
Seizures	4 (30.8%)
Nystagmus	3 (23.1%)
Hemiparesis or pain	2 (15.4%)

**Table 2:** Literature review of multifocal DIA/DIGs.

References	Age	Sex	Symptoms	Localization	Extent of surgery	Surgery site	Histology and IHC features	Multifocal status	Adjuvant therapy	Progression	Follow-up	Prognosis
(11)	4 months	Male	Microcephaly, Nystagmus	Suprasellar region; hypothalamus; posterlor fossa;spinal canal	Biopsy	Suprasellar	Anaplasla present, GFAP( +)	Present at diagnosis	Chemotherapy	NA	3B months	Alive
(10)	4 months	Male	Increased intracranial ICP	Suprasellar reglon; diffuse leptomeningeal Spread	Biopsy	Suprasellar	Anaplasia absent, MAP2C(+),GFAP(±),S- 100(±),P53(±)	Present at diagnosis	Vincristine, Cycphosphamide, methotrexate, carboplatine, and etonoside	Yes	5 months	Dead
(13) 2002	2 years	Female	Macrocephaly and vomiting	Right hemisphere, hypothalamic mass, pial and the ependymal	Gross total	Right hemisphere	Anaplasla presents (35%), GFAP(+), Syn(+) Neu(+), Ki67(45%)	Present at diagnosis	Vincristine and carhoplatinum	New occurrence of hypothalamic mass as well as of the pial and the ependymal metastases	11 months	Dead
(14)	14 months	Female	Enlarging head and mild Left hemiparesis	Frontal lobe,C5 7, TIO, TI2,cauuaequina at the level of L3	Subtotal	Frontal lobe	Anaplasla absent,GFAP(+), Vimentine(+), NSE(+), S- 100(+), CD-99(+), Syn(+), Ki 67(<5%)	Present at diagnosis	Cyclophosphamide, vincristine, cisplatinum, and etoposide	Stable	10 months	Alive
(9)	4 months	Male	Irritability, failure to thrive and increasing head circumference	Suprasellar region;outlet of the fourth ventricle	Partial excision, VP	Suprasellar	Anaplasia absent, Ki 67 (l-2%)	Present at diagnosis	No	Suprasellar mass progressed, Multiple new enhancing nodules depictedon the tentorium and brain stem	1 month	Dead
	3 months	Male	Recurrent seizures and bulging fontanelles	Right hemispheric, basal cisterns and in the subarachnoid space, spinal cord	Gross total	Right hemispheric	Anaplasia absent, Ki 67(1-2% up to >5%}	After diagnosis (6 weeks)	Vincristine and carboplatin.temozola mide	Progressed initially, and stable after temozolamide therapy	S months	Alive
(8)	11 years	Male	Stiffness in both legs. Nick pain and visual blackouts	Right middle frontal sulcus;Left anterior frontal cortex;posterior fossa;spinal cord	Subtotal	Right frontal lesion.left frontal lesion	Anaplasia absent Reticulin(+), GFAP(+)	Present at diagnosis	No	No	NA	improved
(7)	5 years	Male	Generalized seizures	Right temporal lesion;cisterna magna;left cerebellar hemisphere	Gross total	Cisterna magna, left cerebellar hemisphere	Anaplasia absent NCAM(+), GFAP(+), Syn(+), EMA(-)Ki67(3%)	Present at diagnosis	Vincristine and carhoplatin	Stable	12 months	Alive
(6)	11 months	Female	Nystagmus	Suprasellar reglon.cerebellar vermis, spinal cord	Gross total	Cerebellar vermis	Anaplasia absent GFAPf(+), MAP2(+), Vimentin(+), S100 (+),BRAF V600E(+) Ki67(2%)	Present at diagnosis	No	Suprasellar tumor progressed, Spinal cord tumor regressed	16 months	Alive
(15)	5.8 months	Male	Myocardic seizures, Irregular respirations with apneic spells, hyperactive	Temporal prepontine cistern, Leptomeningeal seeding In frontal lobes, pial enhancement along the brain stem and C-spine	Subtotal	**-**	NA	Present at diagnosis	No	No	61 months	Alive
(5	8 months	Female	Macrocephaly, regress ion of milestones	Left thalamus, right cerebellum, right parietal cortex, basal cisterns, tentorium, leptomeninges, spinal cord	Biopsy	Right frontal	Anaplasia absent Ki67(0. 5%- 1%) BRAF V600E (-)	Present at diagnosis	Vincristine, cyclophosphamide, etoposide, and cisplatin	Solid lesions regressed, cystic lesions progressed, new Lesion appeared in cerebellum	12 months	Alive
Our case 1 2017	12 years	Female	Seizures	Right and left tentorium, left parasella, right lateral ventricle and left frontal lobe	Gross total	Right and left tentorium, left frontal lobe	Anaplasia absent Syn(+),Neu(+), NF(+), GFAP( + ),O1igo-2(+),SMA(- ),P53(+),ATRX(+),CD34(- ),IDH-1(-), BRAF V600E(+), Ki67(<3%)	Present at diagnosis	No	Stable	2.8 months	Alive
Our case 2, 2017	6 months	Male	Nystagmus	Suprasellar cistern, bilateral cavernous sinus and hippocampus, intraorbital and cisterna magna	Partial	Suprasellar cistern	Anaplasia absent NeuN(-),GFAR(+),Oligo- 2(+),P53(+),ATRX(-),IDH-l(-), BRAF V600E (+), Ki67(5%)	Present at diagnosis	No	Suprasellar tumor regressed, lesion of fourth ventricle outlet disappeared	26 months	Alive

NA, not given; ICP, intracranial pressure; NA, Not given; VP, Ventriculoperitoneal shunt.

### Surgical Management, Outcome and Molecular Histological Analyses

Ten patients (76.9%) in this series received surgical resection for the symptomatic lesions and three patients (23.1%) underwent biopsy. Case 1 has been previously reported by our group member (16), but we updated some opinions and statements. In the case presented by Setty et al (11), the patient received a suprasellar tumor biopsy as well as surveillance for the hypothalamus, posterior fossa, and spinal canal, and the residual tumors remained stable 38 months later. Bock et al (10) reported the patient underwent a biopsy for suprasellar tumor and observation for the diffuse meningeal spread, in spite of chemotherapy, the patient expired at a 5-month follow-up due to progression. In the case reported by De Munnynck et al (13), the patient underwent gross total resection for the right hemisphere tumor, the hypothalamic mass and ependymal lesions received close observation, however, the spectacular growth of the hypothalamic mass as well as of the pial and the ependymal metastases was noted 5 weeks later, the patient died of tumor progression 11 months after initial diagnosis. According to Knapp et al (14), the patient underwent subtotal resection for the frontal lobe tumor and close observation for the spinal lesions, and the leftover tumors remained stable 10 months later following treatment. Darish et al (9) reported a 4-month-old boy who underwent partial resection of the suprasellar tumor and close observation of lesion in the outlet of the fourth ventricle, but the suprasellar tumor progressed 24 days after the surgery and the patient died of respiratory insufficiency and hypothalamic dysfunction 1 week later; the other patient was alive 8 months after gross total resection of the right hemisphere tumors and chemotherapy for the residual basal cisterns and spinal cord lesions. The residual tumors in the case reported by Uro-Coste et al (7) remained stable 12 months after the surgery. Abuharbid et al (6) reported the patient underwent gross total resection for the cerebellar vermis tumor and observation of the suprasellar and spinal cord tumors, during the follow-up of 16 months, the suprasellar tumor progressed but the spinal cord tumors regressed. In the case reported by Jurkiewica et al. (15), the residual tumors were stable 61 months following surgery. In the case presented by Narayan et al (5), the residual solid lesions regressed spontaneously, the cystic tumors progressed and new lesions appeared in the cerebellum.

Based on the postoperative histological investigation, all of the patients were categorized as WHO grade I DIA/DIGs. Histologically, they frequently showed a desmoplastic spindle cell tumor with a densely eosinophilic collagenous substrate (**Figure 1G**, x 200). In this series, only two patients showed suspected anaplasia histological features, De Munnynck et al (13) reported a case with suspected anaplastic component and high Ki-67 proliferation index (45%). Nine patients reported the Ki-67 proliferation data; eight patients (88.9%) had benign histological phenotype with a very low Ki-67 proliferation rate (0.5-5%). Four patients reported the BRAF V600E status, and three of four patients showed BRAF V600E mutation-positive. Other immunohistochemical markers included NSE (+), GFAP (+), Syn (+), and EMA (-) (**Table 2**).

## Discussion

Taratuto et al. reported the first case of desmoplastic infantile astrocytoma (DIA) in 1984 (17) and Vandenberg et al. reported the first of desmoplastic infantile ganglioglioma (DIG) in 1987 (18). The 2016 WHO classification of Nervous system categorized DIA and DIG together (18, 19) and classified as grade I (3). DIA/DIGs are generally solitary and have a favorable prognosis with long-term disease-free survival after surgical resection (20). DIA/DIGs with multifocal lesions are uncommon, and little is known about their clinical features and management.

DIA/DIGs frequently occur as a supratentorial, contrast-enhanced solid tumor with cystic components (4). And the solid component always manifests as hypointense in T1 and T2 sequences, and the cystic part is hypointense and hyperintense in T2 at brain MRI (15). They frequently involved frontal and parietal lobes; the tumors tend to be large with solid components located at the surface of the cerebral cortex (4, 21). However, we found that multifocal DIA/DIGs are not restricted to the supratentorial region, they can also occur in the suprasellar region, posterior cranial fossa and spinal cord. In this series, we found eight patients (61.5%) had tumors in suprasellar or parasellar region, eight patients (61.5%) in the posterior fossa and eight patients (61.5%) in the spinal cord. Multifocal DIA/DIGs may be confused with fibrous meningioma, inflammatory disease, metachronous or metastatic lesions preoperatively due to its multifocal location and no specific radiological characteristics (5). Hence, neurosurgeons should at least be reminded of the possibility of DIA/DIG when confronting multiple intracranial and spinal lesions in infants and young children.

Our study found no obvious differences regarding histological features between multifocal DIA/DIGs and solitary counterparts. Both of them contained reticulin- and collagen-rich areas with spindle fibroblastic elements, DIGs always show neuronal elements whereas DIAs show astrocytic component (4). Besides, the mitotic activity in both solitary and multifocal DIA/DIGs is low with the Ki67 index ranging between 0.5 and 15% (4).

Some research speculated that multifocal DIA/DIGs were metastases from a single tumor *via* the CSF pathway, and they identified tumor cells in the CSF of these patients (5, 9, 11, 13). However, the pattern of direct shedding of tumor cells from the initial tumor into CSF, survival and migration with CSF, followed by distal implantation on the pial surface of CNS was only an assumption in the literature, but was poorly supported by empirical evidence (22). Meanwhile, some authors found that the CSF in multiple DIA/DIGs contained inflammatory cells, chiefly macrophages and signet ring-like cells, but no tumor cells (8, 10). The Case 1 in our series showed the multifocal tumors were located in the brain parenchyma, hinting these lesions were not metastases *via* CSF pathways. Furthermore, twelve patients were found to have multifocal status at the time of diagnosis in this series, excluding the case reported by Darish et al (9), who found multifocal leptomeningeal lesions six weeks after the initial diagnosis of DIA/DIGs. Hence, the mechanism of multifocal DIA/DIGs, metastases, or an inherited genetic disorder, remained to need further study.

Solitary DIA/DIGs were frequently stable and had long relapse-free intervals even after partial resection, hinting at the indolent nature of the tumor (4, 23, 24). But the growth pattern and natural history of multifocal DIA/DIGs were unclear. Despite the WHO I grade and low Ki-67 proliferation rate (0.5%-5%), we found multifocal DIA/DIGs can sometimes be fast-growing after surgical resection and chemotherapy. In this series, 3 patients (27.3%) died of tumor progression and 6 of 13 (46.2%) patients showed residual tumor progression or new lesions occurrence during the follow-up. Hence, long-term careful monitoring is needed. It is worth mentioning that some tumors in three patients spontaneously regressed, and this phenomenon should be taken into consideration when treating multifocal DIA/DIGs.

Gross total resection is always associated with far superior outcomes in solitary DIA/DIG (25, 26). But the treatment of multifocal DIA/DIGs can be more complex due to the multifocal location. It was suggested that surgical treatment can be performed in symptomatic mass to get the histopathological diagnosis and improve quality of life, while close observation for the asymptomatic tumors. As for the adjuvant chemotherapy, despite some studies suggesting that vincristine and carboplatin may be contributing to control DIA/DIGs in the adjuvant setting, but no consensus had reached so far (5, 7, 13). BRAF gene mutation has been found in 43.8% solitary DIA/DIGs recently (27, 28), which hinted DIA/DIGs were another low-grade MAPK pathway-driven neuroepithelial tumor and might allow for the use of targeted molecular therapies, such as vemurafenib (29). In this study, four patients examined the BRAF status, and 3 of 4 patients depicted BRAF V600E mutation, which showed higher occurrence than the solitary counterparts.

Several limitations should be stated in our study. Firstly, due to the limited sample size, it is difficult to conduct a statistical analysis to find the prognostic factors. Secondly, the present study included both institutional series and literature case reports, some intrinsic limitations should attend to. Thirdly, only four patients in this series screened for the BRAF mutation, further multicenter studies were needed to investigate the incidence of BRAF mutation in multifocal DIA/DIGs. Finally, future studies, especially multicenter prospective studies with a large sample size of this rare tumor subset, are needed.

## Conclusions

Despite the benign histology of multifocal DIA/DIGs, they had incompatibly aggressive clinical features and poor prognosis. They can occur in the entire neural axis and some of tumors can undergo spontaneous regression. They should also be included in the differential diagnosis of multiple intracranial and spinal cord tumors in infants and young adults. We suggest that symptom-producing tumors be surgically resected with care, and that residual lesions be followed up closely. Due to the high risk of residual tumor progression, regular long-term follow-up is essential.

## Data Availability Statement

The original contributions presented in the study are included in the article/supplementary material. Further inquiries can be directed to the corresponding authors.

## Ethics Statement

The studies involving human participants were reviewed and approved by the Institutional Review Board of West China Hospital, Clinical data for patients were retrospectively sourced after having gained approval. Written informed consent to participate in this study was provided by the participants' legal guardian/next of kin.

## Author Contributions

QW and JM, conceptualization, methodology, formal analysis, validation, investigation, data curation, writing - original draft, writing - review and editing, and visualization. JC, writing - review and editing, methodology, and funding acquisition. SZ, data curation. XH, QL, and WL, validation and resources. YJ and LS conceptualization, methodology, and validation. All authors contributed to the article and approved the submitted version.

## Funding

This study was supported by the Science and technology project of Sichuan Province (Fund No. 2021YJ0161), the Medical Research project of Sichuan Province (Fund No. Q20042), the Science and technology Project of Tibet Autonomous Region. The central government guides local projects (Fund No. XZ202102YD0032C).

## Conflict of Interest

The authors declare that the research was conducted in the absence of any commercial or financial relationships that could be construed as a potential conflict of interest.

## Publisher’s Note

All claims expressed in this article are solely those of the authors and do not necessarily represent those of their affiliated organizations, or those of the publisher, the editors and the reviewers. Any product that may be evaluated in this article, or claim that may be made by its manufacturer, is not guaranteed or endorsed by the publisher.
